# Ultrafast chemistry

**DOI:** 10.1038/s42004-026-02103-6

**Published:** 2026-07-30

**Authors:** Haiwang Yong

**Affiliations:** 1 Department of Chemistry, University of California San Diego, La Jolla, CA USA; 2 Program in Materials Science and Engineering, University of California San Diego, La Jolla, CA USA

**Keywords:** Physical chemistry, Chemical physics

## Abstract

*Communications Chemistry* is delighted to present a Collection of research articles highlighting recent developments in the field of ultrafast chemistry. Here, Editorial Board Member Professor Haiwang Yong introduces the topic and outlines the themes covered by the Collection.

Ultrafast chemistry concerns chemical and physical processes that unfold on picosecond, femtosecond, and even attosecond timescales, where the motions of nuclei and electrons governing chemical reactions can be observed in real time using time-resolved spectroscopic techniques. A long-standing goal of the field has been not only to monitor chemical reactions at their most fundamental time and length scales, but also to manipulate and control these processes with comparable precision to direct reaction outcomes.

The origins of ultrafast chemistry can be traced to early efforts to capture transient intermediates in reaction mechanisms. A major breakthrough came with the development of flash photolysis^1^, recognized by the 1967 Nobel Prize in Chemistry awarded to Ronald George Wreyford Norrish, George Porter, and Manfred Eigen for studies of extremely fast chemical reactions using short pulses of energy. Flash photolysis established the conceptual foundation for pump–probe spectroscopy, in which a chemical process is initiated by a pump pulse and monitored by a delayed probe pulse. Initially limited to microsecond and nanosecond timescales, advances in laser technology, notably chirped pulse amplification^2^ in the 1980s, recognized by the 2018 Nobel Prize in Physics, enabled intense ultrashort laser pulses and pushed time resolution into the femtosecond regime, giving rise to femtochemistry^3^. This was recognized by the 1999 Nobel Prize in Chemistry awarded to Ahmed Zewail, whose pioneering work enabled direct observation of atomic motions during chemical reactions.

The emergence of attosecond light pulses^4^, recognized by the 2023 Nobel Prize in Physics awarded to Pierre Agostini, Ferenc Krausz, and Anne L’Huillier, has extended this frontier into attochemistry, where electron motion in molecules and materials can be tracked with unprecedented temporal resolution. Today, ultrafast chemistry is a highly interdisciplinary field focused on fundamental processes such as charge and energy transfer, proton transfer, isomerization, bond rearrangement, and nonadiabatic relaxation in increasingly complex molecular, condensed-phase, and interfacial environments. Progress has been driven by advances in ultrafast sources, such as high-harmonic generation, X-ray free-electron lasers, and relativistic electron pulses, alongside developments in ab initio electronic structure theory and molecular dynamics simulations, which are essential for interpreting experiments and predicting ultrafast processes.

This Ultrafast Chemistry collection reflects the breadth and rapid evolution of the field through three interconnected themes: chemical and photo-induced dynamics; charge and energy transfer; and ultrafast tracking, transients and intermediates. The studies highlighted below represent selected examples that illustrate broader directions in the field; together with the full collection, they highlight how ultrafast chemistry continues to advance from observing elementary processes toward predictive and ultimately controllable frameworks for chemical dynamics.

## Chemical and photo-induced dynamics

Chemical and photo-induced dynamics can be strongly affected by chemical environment, with gas-phase, condensed-phase, and interfacial studies each offering distinct and complementary insights. Ultrafast studies in the gas phase provide fundamental insight into the quantum dynamics underlying photochemical and photophysical processes in molecules under well-defined conditions, free from environmental perturbations and many-body interactions. For example, Matselyukh et al. report a 1.5 fs delay during population transfer between two intersecting quantum states in CF_3_I^+^ when coupling to additional states is present, whereas such transfer is expected to be instantaneous in a strict two-state system (10.1038/s41467-025-62162-6)^5^. In another study, Lam et al. fully characterize the photochemical reaction pathways associated with various fragmentation channels of nitrobenzene following UV excitation using time-resolved Coulomb explosion imaging and covariance mapping (10.1038/s42004-025-01672-2)^6^. Moving from observation toward rational design, Hymas et al. demonstrate strategies for optimizing photon conversion pathways by modulating nonradiative decay through steric and electronic substitutions in cinnamate derivatives in both the gas and solution phases (10.1038/s42004-026-01963-2)^7^.

In condensed-phase and interfacial systems, chemical dynamics are profoundly impacted by the surrounding environment. Solvent reorganization and intermolecular motion can actively reshape excited-state landscapes and even participate directly in chemical reactions. For example, Jarupula et al. reveal ultrafast solvent-to-solute proton transfer mediated by coherent intermolecular vibrations, emphasizing the active mechanistic role of surrounding solvent molecules (10.1038/s42004-026-01917-8)^8^. Condensed-phase photochemistry offers further opportunities for rational molecular design, as demonstrated by Garrow et al., whose study of azanaphthalenes links competing excited-state relaxation pathways to tunable photoactive functionality (10.1038/s42004-024-01403-z)^9^. Moving toward interfacial systems, where ultrafast chemistry intersects with heterogeneous catalysis and functional materials, Gleissner et al. propose an O_2_–TiO_2_ charge transfer complex responsible for initiating the oxygen activation pathway in the oxidation of CO to CO_2_ on rutile TiO_2_, showing that oxidation dynamics on rutile are faster despite anatase being the more active photocatalyst (10.1038/s42004-026-01901-2)^10^. Jordan et al. reveal that the hydrated electron at the water–air interface exhibits relaxation dynamics distinct from those in bulk water (10.1038/s41467-023-44441-2)^11^. These studies point to the growing role of ultrafast measurements in understanding photochemical dynamics under realistic heterogeneous conditions.

## Charge and energy transfer

A central question in ultrafast chemistry centers on how charge and energy flow through matter on their natural timescales. Charge and energy transfer can occur on very short timescales and are usually accompanied by complex dynamical pathways. Such processes are fundamental to many chemical reactions and underpin applications ranging from natural photosynthesis to artificial light-harvesting systems and photocatalytic processes. For example, Fields et al. show that orbital coupling can greatly accelerate the escape of a photoexcited electron from an argon atom confined within a fullerene cage, compared with the isolated atom (10.1038/s41467-025-60260-z)^12^. In aqueous solutions, Muchová et al. probe the attosecond formation of charge-transfer-to-solvent states in hydrated ions, revealing the associated ultrafast electron dynamics on sub-femtosecond timescales (10.1038/s41467-024-52740-5)^13^. Charge transfer can often be coupled with proton transfer and spin transitions. Yuan et al. use photodetachment photoelectron spectroscopy to directly observe proton-coupled electron transfer (PCET) in hydrogen-bonded phenolic nitrate complexes, in which a much slower rising edge provides spectroscopic evidence of PCET (10.1038/s42004-024-01257-5)^14^. In another study, Nakamura et al. show that photoinduced charge transfer can trigger ultrafast spin conversion in cobalt–tungstate molecular photomagnets (10.1038/s41467-025-60401-4)^15^. Jaiswal et al. reveal sub-100-fs energy transfer in coenzyme NADH mediated by a charge-transfer state, providing insights into coherent excitation energy transfer processes in stacked multichromophoric aggregates such as DNA strands (10.1038/s41467-024-48871-4)^16^.

## Ultrafast tracking, transients and intermediates

This theme highlights the rapid development of both theoretical and experimental techniques in today’s ultrafast chemistry field, enabling ultrafast tracking of chemical dynamics, such as transient states and intermediates, with a comprehensive understanding from both energetic and structural perspectives. State-of-the-art computational chemistry methods are becoming increasingly powerful for predicting reaction pathways that remain difficult to observe in detail experimentally. For example, Jia et al. use nonadiabatic molecular dynamic simulations to model optically controlled phase transitions from graphite to diamond, revealing how specific phonon modes regulated by different laser parameters can influence ultrafast structural phase transitions (10.1038/s41467-025-67064-1)^17^. In parallel, the continued development of advanced ultrafast spectroscopic methods provides unique sensitivity to electronic and vibrational dynamics. Barlow et al. use femtosecond X-ray absorption spectroscopy to track nuclear motion in a manganese-based tri-nuclear single-molecule magnet, resolving small bond-length changes associated with magnetically active Jahn–Teller mode (10.1038/s41467-024-48411-0)^18^. Ultrafast diffraction and scattering approaches further add spatial resolution, enabling transient structures to be captured in real space and real time. Jiang et al. use super-resolution femtosecond electron diffraction to visualize molecular dynamics at conical intersections during a ring-opening reaction, uncovering a 30-fs timescale for the molecule to traverse between two conical intersections (10.1038/s41467-025-61975-9)^19^. Schori et al. capture the structural dynamics of the photodissociation of iron pentacarbonyl using ultrafast X-ray scattering, offering mechanistic insights into transition metal catalytic systems (10.1038/s41467-025-60009-8)^20^. Moreover, Goluben and Hassan present an interesting perspective on quantum attomicroscopy, discussing the potential of applying this technique to image charge migration in DNA base pairs (10.1038/s42004-026-02080-w)^21^.

## Outlook

Looking ahead, the future of ultrafast chemistry appears to unfold along two closely connected directions. The first is the pursuit of a complete understanding of dynamics in increasingly complex chemical environments. This will require continued integration of complementary ultrafast spectroscopic, imaging, and theoretical approaches capable of connecting experimental observables to chemical dynamics and kinetics across different spatial and temporal scales. Meanwhile, the emergence of attochemistry points toward an additional frontier at the shortest timescales, where the ability to probe and manipulate coherent electronic motion may open fundamentally new directions in ultrafast chemistry, as discussed in detail by Calegari and Martin in their Comment article on open questions in attochemistry (10.1038/s42004-023-00989-0)^22^. The second direction is to move beyond observation toward active control. While the examples in this collection demonstrate increasingly detailed access to ultrafast chemical dynamics, the long-term goal is to selectively manipulate atomic and electronic motions to direct chemical outcomes on demand. As ultrafast techniques continue to advance in sensitivity and applicability to realistic systems, the prospect of rationally steering chemical reactions on their intrinsic timescales becomes possible. In this sense, the future of ultrafast chemistry lies not only in observing ever more complex dynamics with greater fidelity, but also in developing the capability to predict, design, and control chemical reactions at their most fundamental level.
